# Pharmacogenomic diversity among Arab populations: A systematic review

**DOI:** 10.1016/j.isci.2026.115191

**Published:** 2026-02-28

**Authors:** Zeina N. Al-Mahayri, Mais N. Alqasrawi, Lubna Q. Khasawneh, Sahar M. Altoum, Areej S. Albawa’neh, Lilas Dabaghie, Bassam R. Ali

**Affiliations:** 1 Department of Biomedical Sciences, College of Health Sciences, Abu Dhabi University, Al Ain, UAE; 2 Department of Genetics and Genomics, College of Medicine and Health Sciences, United Arab Emirates University, Al Ain, UAE; 3 Pharmacy Department, Sheikh Tahnoon Bin Mohammed Medical City, Al Ain, UAE; 4 Research and Graduate Studies Department, Mohammed Bin Rashid University of Medicine and Health Sciences, Dubai Health, Dubai, UAE

**Keywords:** Health sciences, Medicine, Clinical genetics, Human genetics

## Abstract

Pharmacogenomics enables precision pharmacotherapy by linking genetic variation to drug response, yet Arab populations are underrepresented in global reference datasets. We systematically synthesized pharmacogenomic allele-frequency evidence across Arab countries, focusing on clinically actionable genes, to describe population variation, identify high-priority variants, and highlight research gaps. We analyzed 295 studies including 94,346 individuals from 19 countries, pooled country-level allele counts for frequently tested variants, and compared pooled estimates with Middle Eastern reference frequencies. Across most loci, allele-frequency profiles were broadly similar between countries, but several variants showed marked, locus-specific differences with direct relevance to anticoagulants, statins, thiopurines, antidepressants, and fluoropyrimidines. Evidence was uneven across countries and often limited by inconsistent genotyping and incomplete reporting of haplotypes and structural variation. These findings support variant-focused implementation, underscore the need for better population coverage and standardized reporting, and motivate development of a regional pharmacogenomics resource to improve the safety and effectiveness of therapy.

## Highlights

• Synthesized pharmacogenomic allele frequencies from 295 studies across 19 Arab countries
• Most actionable variants show similar frequencies with locus-specific differences
• Pooled analyses identify deviations from gnomAD-ME for key drug-response variants
• Supports variant-focused implementation and regional pharmacogenomics resources

## Graphical abstract

**Figure:**
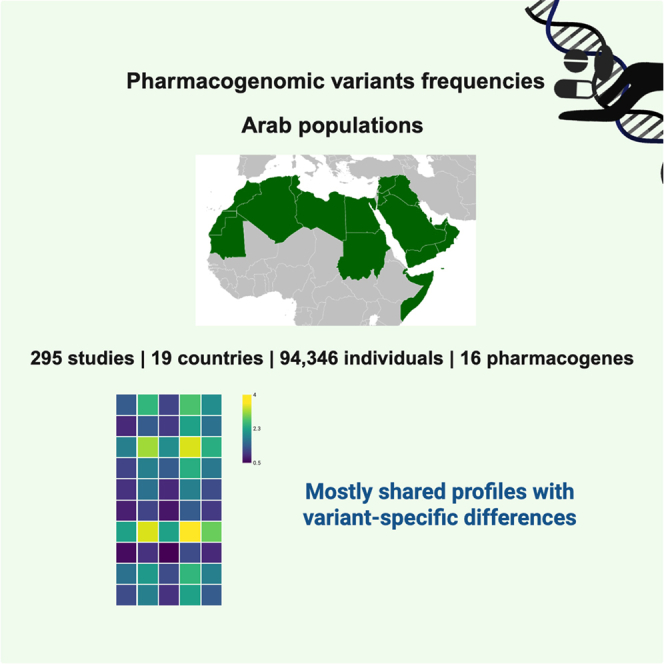

## Introduction

Adverse drug reactions (ADRs) significantly impact global healthcare, ranking as a leading cause of hospitalization and mortality worldwide. Genetic variants are crucial in influencing drug efficacy and the risk of ADRs, especially for commonly prescribed medications.^1^ Recognition of genetic contributions to drug responses spurred the development of pharmacogenetics as a distinct scientific field. With advances in sequencing technologies, pharmacogenomics emerged, providing a comprehensive framework for evaluating genetic determinants of drug effectiveness and safety. By leveraging genomic data, pharmacogenomic research aims to optimize drug therapy, enhancing treatment effectiveness while minimizing adverse effects, thus moving beyond the traditional “one-size-fits-all” medical approach toward personalized medicine.^2^

Pharmacogenetic research has undergone substantial evolution since its inception in the 1950s. Based on familial and twin analyses, early studies established a genetic basis for differences in drug metabolism and adverse reactions. Subsequent technological advancements facilitated the transition from candidate gene studies, focused on single-gene effects, to genome-wide association studies (GWASs), enabling comprehensive exploration of genetic influences on drug responses.^3^ Currently, pharmacogenomics integrates multi-dimensional omics data, leveraging large biobanks and sophisticated bioinformatics tools, to identify, validate, and mechanistically elucidate complex gene-drug interactions, propelling the field toward personalized pharmacotherapy.^3^^,^^4^

Comparative analyses of genetic variation across populations have revealed significant differences in variant frequencies, leading to the development of population pharmacogenomics. This field underscores the importance of considering population-specific genetic profiles when implementing precision medicine.^5^ Understanding genetic diversity across populations is sought to enhance public health strategies and clinical outcomes at the population level.^6^ Moreover, it is well recognized that underrepresented populations constitute rich reservoirs of rare genetic variants that may elucidate complex traits, including differential responses to drugs. Exploring these unique genetic variants can substantially enhance the precision and efficacy of pharmacological studies and has the potential to optimize drug therapies globally.^7^

Within this context, Arabs represent approximately 5% of the global population and encompass remarkable diversity in genetic structure and health considerations, reflecting their varied geographical origins, ethnic backgrounds, and countries of residence. Spanning the Arabian Peninsula, the Levant, and North Africa, the Arab world includes countries with significant economic disparities, ranging from some of the wealthiest to the most economically challenged nations. Genetically, present-day Arabs exhibit substantial evidence of admixture. Yet, they also possess distinct genetic signatures and face unique risk factors for common diseases,^8^ emphasizing the necessity of tailored pharmacogenomic insights.

Despite the increasing recognition of pharmacogenomics’ role in personalized medicine, research efforts in the Arab region remain fragmented, and pharmacogenomic testing is not widely integrated into clinical practice. Some studies have mapped the allele frequencies of key pharmacogenes, revealing distinct genetic variations that influence drug response among Arabs. However, many of these studies are limited in scope, small-scale, or focused on specific subpopulations, and comprehensive regional databases or standardized clinical guidelines remain absent.^9^^,^^10^^,^^11^^,^^12^^,^^13^

Consequently, a structured and comprehensive assessment is critically needed.

To address this gap, the present study systematically retrieves, analyzes, and synthesizes published pharmacogenomic data from Arab populations. It provides a structured evaluation of existing research, highlights emerging trends, identifies research gaps, and suggests critical areas requiring further investigation. While curated resources such as ClinPGx aggregate pharmacogenomic associations and guideline annotations, they do not provide a systematic, region-specific synthesis of primary studies, nor do they assess population coverage, study heterogeneity, or research gaps across Arab countries.

## Results

### Study flow

Using 39 search words in all possible “gene, country” combinations (Table 1) in the six searched databases and registries, we retrieved 129,399 reports.

**Table 1:** Search terms including genes with clinical annotation guidelines (A) and Arab Countries (members of the Arab League) (B)

Genes with clinical annotation guidelines	Arab Countries
*CYP2D6* *CACNA1S* *RYR1* *CYP2C19* *DPYD* *NUDT15* *TPMT* *IFNL3* *SLCO1B1* *ABCG2* *CYP3A5* *CYP2B6* *UGT1A1* *CYP4F2* *VKORC1* *CYP2C9*	Algeria Bahrain Comoros Djibouti Egypt Iraq Jordan Kuwait Lebanon Libya Mauritania Morocco Oman Qatar Palestine Saudi Arabia Somalia Sudan Syria Tunisia United Arab Emirates (UAE) Yemen

**Figure 1:**
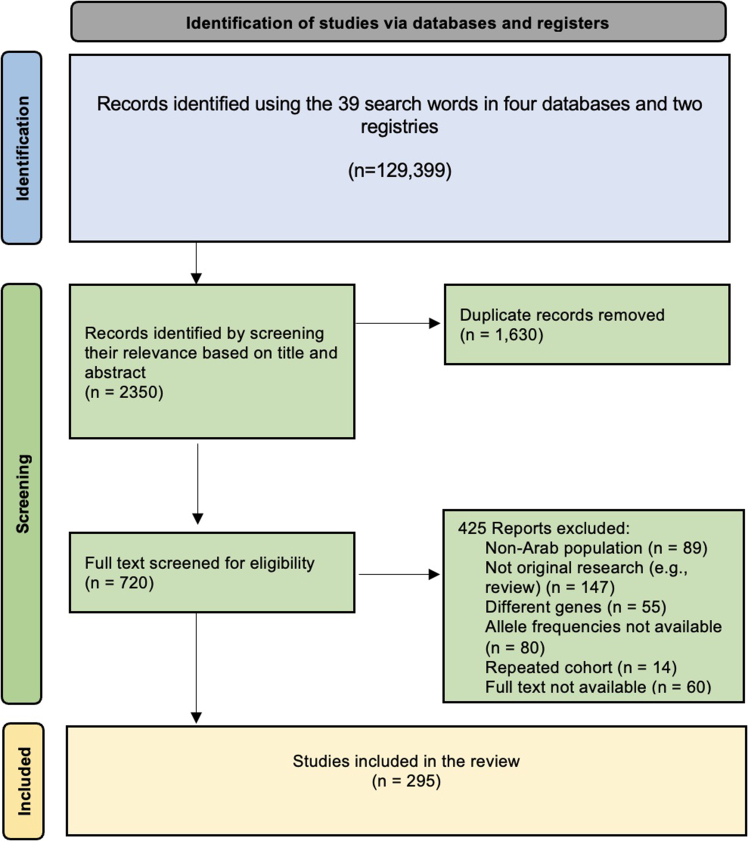
Systematic review study selection process: PRISMA flowchart

The title and abstract screening of these reports yielded 2,350 results, of which 720 were retained after removing duplicates. The full-text screening process resulted in 295 original studies. The details of exclusion and inclusion are illustrated in Figure 1. (The Preferred Reporting Items for Systematic Reviews and Meta-Analyses (PRISMA) checklist is attached in the supplementary material).

### Included studies

The 295 studies included in this review were conducted on cohorts from 19 countries. The total number of participants in the included studies summed up to 94,346 participants. Egypt had the most significant number of pharmacogenetic studies (108 studies), followed by Saudi Arabia (38 studies) and Jordan (37 studies). We were unable to identify pharmacogenomics studies conducted in the Comoros, Djibouti, and Mauritania. Moreover, the most significant number of studied pharmacogenetic variants were retrieved from studies conducted in Saudi Arabia (223 variants), Egypt (193 variants), and Qatar (179 variants). Table S1 lists all variants retrieved from the included studies listed per country, with the counts of studies, number of participants per country, and variants per country.

### Frequencies of variants in individual genes

The complete list of retrieved variants and their frequencies was sorted according to the gene in which they are located (Table S2) and examined for significant findings. For the results of the comparison of pooled allele frequencies with gnomAD data, all test statistics, raw and adjusted *p*-values, and the direction of differences (higher/lower MAF-study relative to gnomAD-ME) are reported in Table S3. Beyond reporting pooled allele frequencies, this analysis evaluates cross-study consistency, population representation, and discrepancies between national studies and global reference datasets, enabling identification of evidence gaps that are not apparent from database records alone. The following paragraphs list the main findings for each gene.

#### ATP-binding cassette superfamily G member 2

The ATP-binding cassette superfamily G member 2 (*ABCG2*) encodes a protein active in drug efflux transport.^14^ Twelve studies from seven countries examined *ABCG2* in Arab populations. The most studied variant in this gene was *ABCG2*: *rs2231142* (C421A), which was studied in six countries. The two highest frequencies of this variant were reported in Egypt^15^ and Saudi Arabia,^16^ where it occurred at frequencies of 0.27 and 0.21, respectively. This variant has been associated with the lipid-lowering drug rosuvastatin, response, and pharmacokinetics.^14^^,^^17^ According to gnomAD (https://gnomad.broadinstitute.org/), the Middle Eastern frequency of the alternative allele is 0.0569. Notably, most studies reporting higher frequencies were conducted in disease-specific cohorts, predominantly among patients with cancer, whereas the single study conducted in healthy volunteers reported a frequency (0.061) comparable to gnomAD.^11^ To reduce bias from heterogeneous and underpowered cohorts, allele counts were pooled for Egypt, the population with the most comprehensive available data, and compared with gnomAD using a chi-square test, which confirmed a statistically significant difference (χ^2^ = 53.03, *p* < 0.00001). Nevertheless, given the limited sample sizes, disease-enriched study designs, and inconsistent findings across populations, these observations should be interpreted cautiously, and rs2231142 should be primarily considered for pharmacogenetic relevance rather than disease susceptibility.

#### *CACNA1S* and *RYR1*

*RYR1* encodes a ryanodine receptor isoform 1 protein, a component of the calcium release channel. While *CACNA1S* encodes the α1s subunit of the calcium channels dihydropyridine receptor (DHPR), which is predominantly found in skeletal muscle, it plays a vital role in muscle contraction. Mutations in both genes are linked to several myopathies and the pharmacogenetic disorder malignant hyperthermia, a side effect of volatile inhalational anesthetics.^18^ Our search revealed that variants of these two genes were studied only in Qatar.^19^^,^^20^^,^^21^ Only two *CACNA1S* variants were detected in these studies, *rs12139527*^19^ and *rs3850625*.^20^ The same two variants were reported in the malignant hyperthermia susceptibility (MHS)-UK cohort, as well as in other international MHS cohorts.^22^ On the other hand, the *RYR1*-reported variants were all largely previously unreported in Arab cohorts, except for *RYR1*: c.*1589G*>A, one of the variants designated as a causative variant by the European Malignant Hyperthermia Group (EMHG).^18^

#### CYP2B6

Ten studies explored *CYP2B6* variants across six Arab countries. The most frequently studied variant was *rs3745274* (*516G*>T). Across most populations, the reported MAF ranged between 0.20 and 0.31, consistent with the Middle Eastern frequency reported in gnomAD (MAF = 0.278). However, one study from Morocco reported a substantially higher MAF (0.553).^23^ Using pooled allele counts, statistical comparison with gnomAD revealed significant deviations in some populations, with Morocco showing a significantly higher frequency (χ^2^ = 184.6, *p* < 0.0001) and both Jordan (MAF = 0.20; χ^2^ = 7.21, *p* = 0.02) and Saudi Arabia (MAF = 0.215; χ^2^ = 6.34, *p* = 0.03) showing significantly lower frequencies relative to gnomAD, while no significant differences were observed for Egypt, Lebanon, Qatar, or the UAE. Although rs3745274 is a core variant shared across multiple *CYP2B6* star alleles (including ∗6 and ∗9), most included studies reported this variant in isolation without phased haplotype resolution or assessment of additional defining variants. Therefore, analyses were performed at the single-variant level rather than the star-allele level to avoid misclassification. This variant has a robust clinical association with the metabolism, toxicity, and dosage adjustments of efavirenz, the HIV antiviral medication, and the metabolism of the antidepressant sertraline^24^

#### CYP2C9

Forty-five studies genotyped *CYP2C9* variants and reported the frequencies of 38 variants from populations in 13 Arab countries. The most extensively studied variant in this gene is *rs1799853* (*430C > T*), which determines the *CYP2C9*∗2 allele. Across most Arab populations, the MAF of this variant ranged between 0.11 and 0.14, comparable to the Middle Eastern frequency reported in gnomAD (MAF = 0.121). However, pooled allele-count-based analysis revealed significantly higher frequencies in Tunisia (MAF = 0.179; χ^2^ = 25.6, *p* < 0.001)^25^^,^^26^ and Saudi Arabia (MAF = 0.136; χ^2^ = 8.84, *p* = 0.009),^27^^,^^28^^,^^29^ while no significant differences were observed in the remaining populations.

**Figure 2:**
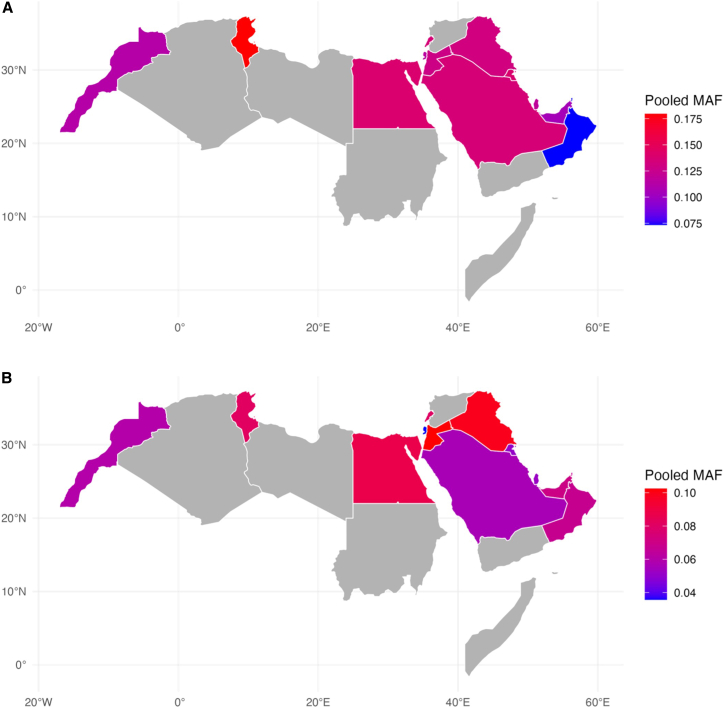
Geographic map showing the minor allele frequencies for two CYP2C9 variants in some Arab countries (A) CYP2C9∗2. (B) CYP2C9∗3.

Another commonly tested variant is *rs1057910 (1075A>C),* which defines the *CYP2C9*∗3 allele. Most Arab populations exhibited MAFs close to the gnomAD Middle Eastern reference (MAF = 0.073). Statistically significant deviations were observed in Jordan, where the allele frequency was higher (MAF = 0.103; χ^2^ = 11.4, *p* = 0.003),^30^^,^^31^ and in Qatar and Saudi Arabia, where lower frequencies were detected (MAF = 0.052 and 0.056, respectively; both *p* < 0.001).^12^^,^^27^ Figure 2 depicts descriptive pooled-MAF across countries, while statistical significance was assessed using pooled allele counts and is reported in Table S3.

Besides these two commonly studied variants, multiple rare variants have been examined and reported in *CYP2C9*, particularly in studies using next-generation sequencing (NGS) rather than selective genotyping. One significant variant, *rs7900194*, which defines *CYP2C9*∗8 and decreases the enzyme activity and affects warfarin clearance,^32^ was reported in Arabian Peninsula populations such as Omanis (MAF = 0.07),^33^ Saudis (MAF = 0.005),^27^ and Qataris (MAF = 0.02).^34^ Notably, the same allele is frequently observed in individuals of African ancestry (MAF = 0.047).^32^^,^^35^

#### CYP2C19

Fifty-seven studies investigated *CYP2C19* variants among Arabs from 14 different countries. The most studied variants identified the star alleles ∗2, ∗3, and ∗17. The highest MAFs of the *CYP2C19*∗2 variant (*rs4244285/681 G>A*) were reported from Sudan (MAF = 0.358),^36^ Jordan (MAF = 0.35,^37^ 0.34^38^), and Saudi Arabia (MAF = 0.322).^39^ Several populations, such as Sudan, displayed apparently high allele frequencies in individual studies; however, these estimates were derived from single studies with limited sample sizes and were therefore not included in pooled or inferential analyses and should be interpreted cautiously.

Nevertheless, other studies from the same populations showed lower frequencies of the same allele. In Jordan, four different studies with a total sample size of 1,242 reported close frequencies of the ∗2 allele (MAF range 0.08–0.09).^10^^,^^40^^,^^41^^,^^42^ Similarly, two large studies from Saudi Arabia with samples of 11,889 and 768 indicated lower MAFs of this allele (0.096 and 0.076, respectively).^27^^,^^28^ However, when allele counts were pooled across eligible studies and compared with gnomAD Middle Eastern data, statistically significant differences were observed only in specific populations. Pooled analyses demonstrated significantly higher MAFs in Iraq, Jordan, and Morocco compared with gnomAD-ME (FDR-adjusted *p* < 0.05), whereas pooled frequencies in other populations, including Saudi Arabia, Qatar, and Egypt, were comparable to the reference dataset.

A similar pattern was observed for the *CYP2C19*∗3 allele (rs4986893/636G>A). While most populations exhibited very low pooled MAFs consistent with gnomAD-ME (MAF ≈0.001), significantly higher frequencies were detected in Egypt, Iraq, Morocco, and Saudi Arabia after pooling allele counts (FDR-adjusted *p* < 0.05). Several reports describing extremely high frequencies in small cohorts were inconsistent with pooled estimates and global reference data and were therefore not interpreted as representative of population-level frequencies.

**Figure 3:**
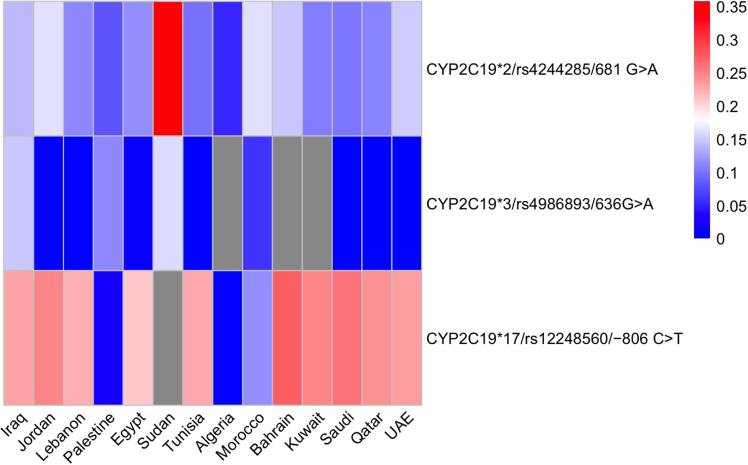
Pooled minor allele frequencies (MAFs) of CYP2C19∗2, 3, and 17 across Arab populations Gray cells indicate missing pooled data.

Nineteen studies conducted in 12 populations reported the frequencies of rs12248560/−806 C>T, which determines the *CYP2C19∗17* allele. Pooled allele frequencies were generally similar to those reported in gnomAD-ME across most Arab populations. A statistically significant (*p* < 0.05) lower frequency was observed in Morocco, Algeria, and Palestine relative to gnomAD-ME. Figure 3 summarizes the pooled MAF distributions of the three most commonly studied *CYP2C19* variants across Arab populations.

#### CYP2D6

Thirty-eight studies examined *CYP2D6* in Arab populations originating from 13 countries. This gene encodes up to 5% of hepatic cytochrome P450 enzymes and is involved in metabolizing 25% of prescribed medications.^43^ Hence, it has been repeatedly studied and reviewed in Arab populations, although our search strategy retrieved more extensive data than the latest efforts.^44^ The full list of studies and the retrieved variants is listed in Table S2.

**Figure 4:**
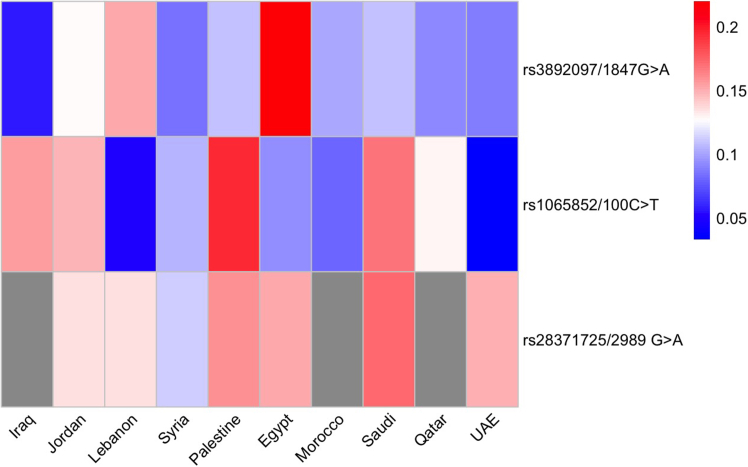
Pooled minor allele frequencies (MAFs) of the three most studied CYP2D6 variants per country Gray cells indicate missing pooled data.

The most frequently reported *CYP2D6* loci included rs3892097, the defining variant of *CYP2D6∗4*, rs1065852, a reduced-function variant that contributes to multiple *CYP2D6* haplotypes (including ∗10), but does not uniquely define any single star allele, and rs28371725, associated with *CYP2D6*∗41. The frequencies of these variants are illustrated in Figure 4. [Variants reported in single studies or lacking consistent genotyping across populations were excluded from Figure 4 to ensure comparability and avoid overinterpretation of sparse data.]

The variant *rs3892097*, associated with loss of *CYP2D6* function,^45^ showed its highest reported frequencies (0.22–0.3) across studies from Egypt,^46^^,^^47^ exceeding the corresponding Middle Eastern frequency reported in gnomAD (0.099) by more than 2-fold. Pooled allele-count analysis further indicated significantly higher frequencies of rs3892097 in Egyptian and Lebanese populations relative to gnomAD-ME.

Structural variation at the *CYP2D6* locus was less frequently investigated. Six studies have examined the complete deletion of *CYP2D6*, also known as ∗5; two were conducted in Egypt and detected this variant in MAFs of 0.04 and 0.047.^48^^,^^49^ According to the ClinPGx (formerly PharmGKB) *CYP2D6* information tables, these frequencies are higher than those reported by European populations (0.029) (https://www.clinpgx.org/page/cyp2d6RefMaterials). In addition, a study from Saudi Arabia reported CYP2D6 gene duplication in 21 of 101 individuals.^50^ Other studies have reported active CYP2D6 gene duplications in Arab populations, with particularly high frequencies observed in Algeria^51^ and variable prevalence across the region. Due to limited, heterogeneous screening strategies and, in some studies, the inability to unambiguously assign the duplicated allele, structural variants could not be quantitatively pooled across populations.

#### CYP3A5

Forty-five studies reported the frequency of rs776746, determining the *CYP3A5*∗3 allele. Of these, 29 reported a prevalence exceeding 80%. Lebanon has the highest MAF for this deactivating allele.^52^ Using pooled allele counts and a formal statistical comparison with gnomAD-ME, rs776746 frequencies were significantly lower in most Arab populations than in gnomAD-ME (MAF = 0.905, FDR-adjusted *p* < 0.05), with the exception of Lebanon, where the pooled frequency was comparable. Nevertheless, the less common alleles, *CYP3A5*∗6 and ∗7, have also been studied and reported in multiple Arab populations. For instance, a study from Saudi Arabia reported the MAF of ∗6 and ∗7 as 0.024 and 0.004, respectively.^27^ These alleles interact with the immunosuppressant, tacrolimus, slowing its metabolism and increasing the chances of response in transplant patients compared to wild-type allele carriers.^53^ In contrast to all other pharmacogenetic variants, the lack of carrying any of these variants or having at least one wild-type allele is considered clinically actionable. Hence, some studies reported the ∗1 allele frequencies rather than the alternative alleles,^12^ which complicated the comparative analysis of *CYP3A5* variant frequencies.

#### CYP4F2

Twenty-one different studies evaluated the variants of *CYP4F2* in 13 countries. The most studied variant was rs2108622 *(297G>A),* which identifies the ∗3 allele. The MAF of this variant in the studied populations ranged from 0.3 to 0.5. This allele is more common among Middle Eastern populations than other populations in gnomAD (MAF = 0.38 and 0.19 for Europeans). Our curated data reported a higher frequency (approximately 50% prevalence) in Qatar,^19^ Oman,^33^ and the UAE.^11^ Using pooled country-level allele counts, statistically significant enrichment of CYP4F23 relative to gnomAD-ME was observed in Tunisia, Qatar, Oman, and Saudi Arabia (FDR-adjusted *p* < 0.05). The most significant interaction of this variant is with warfarin dosing. In a systematic review of warfarin-dosing algorithms for various populations, 11 algorithms were applicable for Arab populations (9 from Egypt, one from Sudan, and another from Oman). However, none of these algorithms considered the effect of *CYP4F2* on warfarin dosing.^54^

#### DPYD

This gene, which encodes for dihydropyridine dehydrogenase, is a large pharmacogene spanning 920 kb.^55^ Many studies that examined this gene used NGS and identified multiple rare variants. The complete list of detected variants is provided in Table S2, while Table 2 curates the loss-of-function alleles detected in at least 2 countries. Recently, the Pharmacogenomics Working Group in the Association of Molecular Pathology (AMP) released a joint consensus recommendation for *DPYD* genotyping with other clinical practice committees.^56^ This guideline provides a minimum set of tested variants (Tier 1) and an extended set (Tier 2) to help laboratories prioritize which variants to test in this large gene. Notably, all the variants detected in Table 2 are Tier 1, underscoring the importance of following these recommendations when testing such a large polymorphic gene.

**Table 2:** Minor allele frequencies of loss-of-function variants in *DPYD* in Arab populations

Variant ID	Allele	Sample size	Cohort	Country	Used genotyping method	frequency	Reference (Author, year)
*DPYD∗13/rs55886062*	G	200	100 women with breast cancer +100 controls	Iraq	PCR	0.27	Abbas et al., 2023
	G	181	Solid tumor patients	Saudi Arabia	NGS	0.003	Aboul-Soud et al., 2021
*DPYD∗2A/rs3918290*	A	500	Healthy Volunteers	Jordan	MassARRAY	0.01	Al-Eitan. 2020
	A	200	100 women with breast cancer +100 controls	Iraq	PCR	0.28	Abbas et al., 2023
	A	181	Solid tumor patients	Saudi Arabia	NGS	0.03	Aboul-Soud et al., 2021
*rs115232898*	G	100	healthy volunteers	United Arab Emirates	NGS	0.005	Al-Mahayri et al., 2020
	G	11889	Healthy volunteers	Saudi Arabia	NGS	0.001	Goljan et al., 2022
	G	6045	Healthy Volunteers	Qatar	NGS	0.001367	Jithesh et al., 2022
*rs56038477*	A	11889	Healthy volunteers	Saudi Arabia	NGS	0.005	Goljan et al., 2022
	A	6045	Healthy Volunteers	Qatar	NGS	0.012	Jithesh et al., 2022
*rs67376798*	T	11889	Healthy volunteers	Saudi Arabia	NGS	<0.001	Goljan et al., 2022
	T	6045	Healthy Volunteers	Qatar	NGS	3.22E-04	Jithesh et al., 2022
	T	181	Solid tumor patients	Saudi Arabia	NGS	0.26	Aboul-Soud et al., 2021

#### Interferon Lambda 3

The interferon Lambda 3 (*IFNL3)* gene has been extensively studied in Arab populations. Given the known interaction between *IFNL3* variants and hepatitis progression and therapeutic response, 44 studies were conducted in Egypt, the country with the highest incidence of hepatitis C worldwide.^57^ The most investigated variant was *rs12979860*, with an MAF range of 0.3–0.6, consistent with 0.3 allele frequency reported for Middle Eastern populations in gnomAD. This variant is the only one with a CPIC recommendation to consider testing as a predictor biomarker for PEG-interferon-alpha response.^58^ Nevertheless, 13 other variants were identified in the same gene. Given the high prevalence of hepatitis in the region, there is room for conducting a meta-analysis to evaluate the evidence supporting the association between population-specific IFNL3 variants and hepatitis progression and response to treatment.

#### NUDT15

Ten studies in seven countries reported the MAF of the *NUDT15*∗3 variant (*rs116855232*). Two studies reported the absence of the variant allele in two small cohorts of pediatric patients with ALL from Iraq.^59^^,^^60^ In contrast, it was detected at high prevalence (allele frequencies of 0.035, 0.018, and 0.021) in large cohorts of healthy volunteers (13,473 and 11,889 from Saudi Arabia, and 6,045 from Qatar). Chi-square comparison demonstrated that the frequency reported in these large screening studies was significantly higher than that in Middle Eastern populations in gnomAD (MAF = 0.011; *p* < 0.05). This variant’s importance lies in its interaction with thiopurines, which has led to adding it to the dosing recommendations of thiopurines in oncology.^61^^,^^62^ In general, limited studies have been conducted on this gene in Arab populations. Its higher-than-expected prevalence suggests considering it along with other variants in *NUDT15* for further research.

#### SLCO1B1

Seventeen studies from eight countries investigated the *SLCO1B1* gene. All these studies investigated the prevalence of *rs4149056*, a non-functional allele, while nine other variants were studied sporadically. The *rs4149056* MAF detected was consistent across the examined populations, ranging from 0.142 in Lebanon^63^ to 0.293 in Qatar.^19^ The gnomAD-Middle East frequency (MAF = 0.2) falls within this range. The same variant identifies *SLCO1B1*∗5,∗15, and ∗37 alleles, which are associated with statin pharmacokinetics and toxicity, and has CPIC testing guidelines and statin dosing recommendations.^17^

#### Thiopurine methyltransferase

This gene, which encodes thiopurine methyltransferase, is one of the most extensively studied pharmacogenes across different populations. The curated data was listed in two tables. The first list outlines the outcomes of studies that mention the MAF of specified variants (Table S2). The second (Table S4) lists the outcomes of studies that annotate the most common *TPMT* haplotypes (∗3A, ∗3B, and ∗3C). [Combining this data in the same table was impossible as the ∗3A is determined by the presence of both ∗3B and ∗3C alternative alleles.] Studies in the second table reported the star allele frequency alone without mentioning the individual variant frequency.

Table S4. TPMT star alleles

Despite most studies reporting the rarity of non-functional alleles of *TPMT* among Arab populations, a few studies have reported higher frequencies of *TPMT*∗2 in Iraq,^64^ Egypt,^65^ and Tunisia^66^ that were significantly higher than MAF in gnomAD-ME (MAF = 0.00033; *p* < 0.05).

#### UGT1A1

This gene’s most clinically relevant variants are ∗28, ∗93, and ∗6.^67^ Sixteen of our included studies examined the frequencies of *UGT1A1*∗28, a variant determined by seven (TA) repeats at rs3064744 in the gene’s promoter region. The highest frequencies of this allele were reported from Tunisia, Egypt, and Sudan (0.53, 0.52, 0.49, respectively).^68^^,^^69^^,^^70^ The minor frequencies of the same allele were reported at 0.12 and 0.25 in two studies from Saudi Arabia.^71^^,^^72^

Besides being involved in the glucuronidation of xenobiotics, such as irinotecan, *UGT1A1* also metabolizes bilirubin and other endogenous compounds. The gene variants are associated with Crigler-Najjar and Gilbert syndromes.^73^ Studies that included participants with these syndromes were excluded to avoid skewing the frequencies toward higher-than-expected values in healthy individuals.

#### VKORC1

This gene, which encodes the target of warfarin, an anticoagulant, is one of the most studied pharmacogenes in Arabs, with 47 reports from 15 countries. The most extensively investigated variant is *rs9923231/-1639G>A*, known to have a detrimental effect on gene expression and warfarin dose, and was studied in 37 studies from 14 countries. The highest frequency (MAF = 0.7) of the alternative allele of this variant was reported from Egypt.^74^ Nevertheless, the pooled MAF from all the studies we retrieved from Egypt (*N* = 769) was significantly lower than the MAF in gnomAD-ME (0.53) (χ^2^ = 5.855, *p* = 0.016). Similarly, the pooled MAF was significantly lower in Iraq, Morocco, and Saudi Arabia, while other populations were consistent with the MAF in Middle Eastern populations in gnomAD.

### Collective analysis of the most frequently tested variants

**Figure 5:**
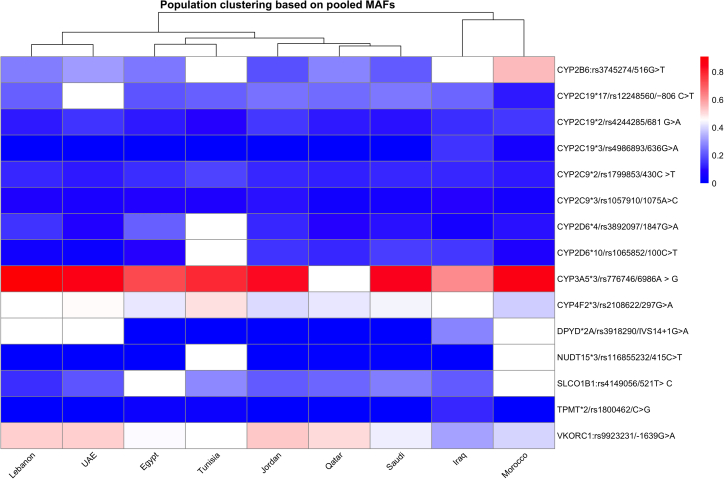
Heatmap with dendrogram illustrating the clustering pattern of populations depending on pooled allele frequencies at selected pharmacogenomic variants gnomAD-ME: Allele frequencies as described in the gnomAD Middle East populations.

The pooled MAF heatmap (Figure 5) showed that the majority of Arab populations exhibit very similar allele frequency distributions across the 15 pharmacogenomic variants examined. Only modest differences were observed for a small number of loci, such as *VKORC1* rs9923231 and *CYP3A5*∗3, whereas most variants displayed comparable frequencies across countries. No strong or consistent geographic clustering patterns emerged, indicating that population-level variation in these clinically relevant pharmacogenes is largely locus-specific rather than region-defined. The heatmap identified a specific variant (*VKORC1*: rs9923231) where the populations exhibit distinct minor allele frequency (MAF) distributions. The variant, affecting warfarin response, shows lower prevalence in Iraq, Morocco, and Saudi Arabia compared to other countries.

**Figure 6:**
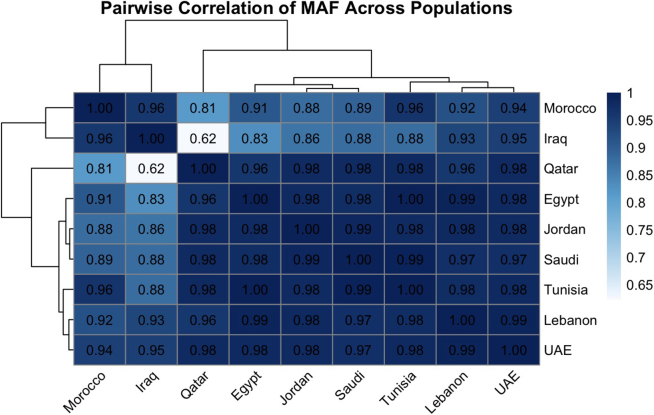
Pairwise clustering of populations depending on pooled allele frequencies at selected pharmacogenomic variants gnomAD-ME: Allele frequencies as described in the gnomAD Middle East populations.

Furthermore, pairwise correlation analysis (Figure 6) showed that most Arab populations share highly similar minor allele frequency profiles across the pharmacogenomic variants included in this review, with correlation coefficients typically exceeding 0.90. Iraq demonstrated moderately lower correlations with some populations (e.g., r ≈ 0.62), reflecting variant-specific differences and a higher proportion of missing data in available studies. Hierarchical clustering of the correlation matrix did not reveal distinct regional groupings; instead, populations clustered closely together with minimal branch separation. Overall, these findings indicate that for the commonly studied pharmacogenes, allele frequency variation across Arab populations is largely homogeneous, with differences primarily driven by individual variants rather than by broad geographic divisions.

## Discussion

The current study provides an extensive and systematic analysis of pharmacogenetic variants in Arab countries. Our analysis revealed meaningful, variant-specific genetic heterogeneity, underscoring the diversity within and between populations, even when studies focus on similar populations. These findings expose considerable gaps in understanding the prevalence of critical pharmacogenetic variants and highlight discrepancies compared to global databases, such as gnomAD. Such gaps, if not bridged with a better understanding, risk the safety and efficacy of pharmacotherapy in the region. Importantly, this systematic synthesis complements curated resources such as ClinPGx by contextualizing database entries within the primary literature, revealing variability in study quality, population coverage, and regional representation across Arab countries.

Our review identified 295 studies from 19 Arab countries, with Egypt, Saudi Arabia, and Jordan contributing the most data. Conversely, no pharmacogenomics studies were identified from Comoros, Djibouti, or Mauritania, and minimal contributions from Algeria, Syria, and Yemen. The uneven representation reflects disparities in research infrastructure and prioritization within the Arab world. Countries with robust research outputs are likely to benefit from better funding, access to genetic technologies, and healthcare systems that support genetic studies. The lack of data from certain countries presents a crucial opportunity for building research capacity through multinational collaborations or regional consortia. Such initiatives could reveal unique genetic variants and enhance the generalizability of findings, thereby improving equity in pharmacogenomics research and, consequently, in pharmacotherapy personalization.

Analyzing the frequencies of common pharmacogenomic variants, notably in *ABCG2*, *CYP2B6, CYP2C9, CYP2D6*, and *CYP3A5*, demonstrated marked intra- and inter-population heterogeneity. For *CYP2D6* in particular, it is important to note that most studies reported single-variant frequencies without phased haplotype resolution or comprehensive copy-number assessment; therefore, these data do not allow reliable inference of *CYP2D6* star-allele distributions or metabolizer phenotypes at the population level. In addition, structural variation at the *CYP2D6* locus, including gene deletions (∗5) and duplications, was sporadically reported but not systematically assessed across populations. The limited and heterogeneous screening approaches used in most studies precluded quantitative pooling of copy-number variation, despite evidence from individual cohorts indicating potentially high prevalence in certain populations. This highlights *CYP2D6* copy-number variation as a critical but under-characterized component of pharmacogenomic diversity in Arab populations.

Similar limitations apply to *CYP2B6*, where rs3745274 was frequently reported without sufficient information to infer star-allele structure. As rs3745274 contributes to multiple *CYP2B6* haplotypes (e.g., ∗6, ∗9), the available data do not allow reliable haplotype-level inference, and variant-based analyses were therefore prioritized to ensure consistency and comparability across studies.

On the other hand, the *ABCG2* rs2231142 variant, which affects rosuvastatin pharmacokinetics and safety, showed higher reported frequencies in several Arab cohorts than those reported in gnomAD-ME. However, these elevated frequencies were predominantly observed in disease-enriched cohorts, particularly among patients with cancer, whereas studies conducted in healthy populations reported frequencies comparable to global reference data. Importantly, these findings should be interpreted in the context of study design and cohort composition, and rs2231142 should be considered primarily for its pharmacogenetic relevance rather than as a marker of disease susceptibility.^40^^,^^75^^,^^76^

Several factors contribute to the observed discrepancies between variant frequencies in Arab cohorts and those reported by gnomAD. Primarily, gnomAD’s data originated from case-control studies focused on common adult-onset diseases without targeted sequencing efforts. Moreover, the local studies we included and gnomAD exhibit significant methodological differences, including variations in sequencing technologies, methodologies, and quality control standards. gnomAD employs stringent quality-control measures and excludes individuals with severe pediatric diseases or those with closely related conditions, aiming to establish a representative dataset of the general population. To mitigate the influence of methodological heterogeneity and underpowered cohorts in the primary literature, we applied a minimum sample size threshold and pooled allele counts across eligible studies within each country prior to statistical comparison. This strategy enabled more reliable population-level estimates while limiting overinterpretation of extreme frequencies derived from small or disease-enriched cohorts. Nevertheless, gnomAD and similar databases tend to underrepresent clinically significant, rare, or previously uncharacterized variants in less-represented populations, such as Arabs, underscoring the need for regional genomic databases that curate data under stringent conditions and adhere to principles of equity and inclusivity. Establishing a regional database would enhance the reproducibility, transparency, and clinical utility of pharmacogenomic research.

Our review identified a scarcity of research in specific areas that warrant focused regional studies. For instance, variants in *CACNA1S* and *RYR1*, linked to malignant hyperthermia, have been studied only in Qatar, with an alarming detection of actionable variants. Although malignant hyperthermia is considered too rare and primarily documented through individual case reports globally, such reports remain scarce in Arab populations.^77^ Consequently, we stress the critical need for anesthetic pharmacovigilance studies in the region, coupled with pharmacogenomic assessments of susceptibility variants, to ensure patient safety and improve outcomes.

Additional research priorities emerged from our systematic review. First, despite the notably high frequencies of *CYP4F2* variants identified among Arab populations, these variants have not yet been incorporated into warfarin dosing algorithms explicitly developed for Arabs. *CYP4F2* plays a modulatory role in warfarin dose requirements by influencing vitamin K metabolism. The rs2108622 variant, which defines the *CYP4F2∗3* allele, results in reduced enzymatic activity and decreased hepatic elimination of vitamin K, thereby increasing vitamin K availability within the coagulation cycle. Carriers of this variant typically require modestly higher warfarin maintenance doses (approximately 5–10%) compared with non-carriers. Multiple studies and meta-analyses support a statistically significant, albeit more minor, contribution of *CYP4F2∗3* to warfarin dose variability relative to *VKORC1* and *CYP2C9*.^78^ While current evidence does not support the routine inclusion of *CYP4F2* in warfarin dosing algorithms, its high prevalence in some populations suggests that its potential contribution may warrant further evaluation in population-specific dosing models.

Second, actionable *DPYD* variants, all Tier 1 variants according to the AMP recommendations, were reported; however, the clinical implications of *DPYD* testing remain underexplored, with a striking absence of region-specific evidence documenting fluoropyrimidine-associated toxicities. Third, given the high prevalence of hepatitis and the substantial frequency of *IFNL3* variants among Arabs, investigating the interactions between *IFNL3* variants and hepatitis disease progression and treatment response constitutes an important area for future research. Lastly, although *NUDT15* variants significantly influence thiopurine metabolism similarly to *TPMT* variants, current data on *NUDT15* in Arab populations remain limited, underscoring the need for expanded research efforts to enhance the precision of thiopurine therapy within this population. Nevertheless, striking discrepancies in reported *TPMT* allele frequencies underscore the need for standardized genotyping methodologies and further research to clarify these conflicting findings.

Analysis of pooled minor allele frequencies across the selected pharmacogenomic variants revealed a high degree of similarity among Arab populations for many of the commonly studied loci. Pairwise correlation and hierarchical clustering analyses demonstrated substantial overlap in allele-frequency profiles across countries, with no consistent or reproducible separation into geographically defined clusters. Together, these findings indicate that pharmacogenomic diversity in Arab populations is characterized by a coexistence of broad similarity and locus-specific divergence, rather than uniform homogeneity or clear geographic stratification. These results differ from regional patterns reported in HLA-focused studies in Arab populations,^79^ likely reflecting the distinct evolutionary and selective pressures acting on immune versus pharmacogenomic loci.

Importantly, the observed heterogeneity was not uniformly distributed across the genome or across populations. Instead, differences were largely driven by specific loci, such as *VKORC1* rs9923231 and *CYP3A5* rs776746, while most other variants showed comparable frequencies across countries. This pattern indicates that pharmacogenomic diversity within the Arab region is best understood as variant-dependent rather than region-dependent, with certain loci exhibiting meaningful population-level variation against a backdrop of overall similarity. These findings provide empirical evidence that challenges simplistic interpretations of Arab populations as either genetically homogeneous or sharply subdivided by geography. While the term “Arabs” is often used as a broad descriptor in genomic research, our results demonstrate that it neither fully captures nor obscures underlying genetic diversity. For widely tested pharmacogenomic variants, population-level differences appear more subtle and context-specific than expected, suggesting that broad implementation strategies may be feasible for many markers, provided that variant-specific exceptions are carefully considered. At the same time, the lack of strong geographic clustering should not be interpreted as evidence of uniformity across Arab populations. The heterogeneity observed across studies reflects differences in cohort composition, study design, sample size, and disease enrichment, underscoring the limitations of relying on underpowered or non-representative datasets. Collectively, these findings highlight the need to move beyond generalized population labels and toward evidence-driven, variant-level evaluation when translating pharmacogenomic knowledge into clinical practice.

Ensuring diversity in pharmacogenomics studies is crucial for making equitable and effective predictions of drug responses. Population-specific variant frequencies have a critical influence on pharmacogenomic outcomes, yet research remains disproportionately Eurocentric. A 2021 review found that 88% of pharmacogenomic GWAS participants were of European ancestry, while non-European groups often carry uncharacterized deleterious variants.^4^ Similarly, 72% of FDA drug trial participants in 2019 were white, reducing the generalizability of findings and contributing to healthcare disparities. Addressing this bias is crucial for advancing precision pharmacotherapy and ensuring equitable therapeutic outcomes.^3^

Challenges hindering the advancement of pharmacogenomics in Arab countries include political instability, underfunding of healthcare research, limited access to advanced genetic technologies, and a lack of awareness among healthcare professionals. Additionally, consanguineous marriages, which are common in Arab societies, contribute to unique genetic profiles that require further study for effective drug dosing and treatment strategies.^9^

Moving forward, coordinated collaborative initiatives are essential for establishing comprehensive, region-specific pharmacogenomic databases, expanding robust population-based studies, and seamlessly integrating genetic testing into clinical practice. Strengthening education, securing targeted research funding, and shaping healthcare policies will significantly facilitate the implementation of pharmacogenomics across Arab nations, thereby enabling more personalized and efficacious therapeutic approaches. We strongly advocate for the creation of a regional pharmacogenomics database that systematically curates genetic variants using stringent quality standards, while ensuring the inclusivity of diverse populations within the region. Such a database would not only directly benefit the local populations it represents but also serve as an invaluable global reference resource, capturing rare genetic variants that could represent potential targets for future drug development.

By harmonizing allele-frequency data, applying minimum sample size thresholds, and performing pooled statistical analyses, this work moves beyond fragmented reports to generate more reliable population-level estimates for clinically actionable pharmacogenes. Our findings demonstrate that, for many commonly tested pharmacogenomic variants, Arab populations share broadly similar allele-frequency profiles, with high correlations across countries and no consistent geographic clustering. At the same time, meaningful differences emerge at specific loci, underscoring that pharmacogenomic diversity in the region is best understood as variant-dependent rather than uniformly region- or population-specific. These results challenge simplistic assumptions of either genetic homogeneity or strict regional stratification among Arab populations and highlight the limitations of using broad population labels without locus-specific evaluation.

Importantly, our findings provide population-specific context that supports the interpretation of entries in curated resources such as ClinPGx. The outcomes revealed gaps in population coverage, methodological heterogeneity, and underrepresentation of several Arab countries. The findings emphasize critical unmet needs, including standardized genotyping approaches, improved reporting of haplotypes and copy-number variation, and expanded population-based studies, particularly for pharmacogenes with complex architectures such as *CYP2D6* and *CYP2B6*.

Collectively, this work provides an evidence-based framework for interpreting pharmacogenomic variation in Arab populations and supports a move toward variant-driven, population-aware implementation strategies. Establishing coordinated regional efforts and high-quality genomic databases will be essential to translating pharmacogenomic knowledge into equitable and effective clinical practice across the Arab world.

### Limitations of the study

Despite its comprehensive scope and rigorous methodological framework, this systematic review has several limitations that should be acknowledged. Firstly, significant heterogeneity existed among the included studies regarding methodological approaches, population selection criteria, and sample sizes, which could affect the accuracy and comparability of reported allele frequencies. Variations in genotyping methodologies might have contributed to discrepancies in variant-detection accuracy and frequency estimates across studies. Secondly, ethnicity was inconsistently reported across the included studies. Although ethnicity information was extracted when explicitly available (Table S2), only a small number of studies provided ethnicity-specific allele frequencies, and reporting was highly inconsistent across populations and genes. As a result, the available data were insufficient to support meaningful aggregation or comparative analyses at the ethnicity level, and analyses were therefore conducted at the country level. Thirdly, due to the limited or absent pharmacogenomic data from several Arab countries, the genetic representation presented here may not fully capture the true extent of regional pharmacogenomic diversity. Finally, this review focused exclusively on pharmacogenes listed with clinical annotations in ClinPGx (formerly PharmGKB), potentially omitting other genes with emerging pharmacogenomic relevance or recently described variants that have not been extensively characterized. Thus, the study might have overlooked additional genetic markers of clinical importance within Arab populations. These limitations collectively underscore the need for more standardized, comprehensive, and inclusive pharmacogenomic research in Arab populations to ensure accurate representation, robust allele frequency data, and effective translation into clinical practice.

## Resource availability

### Lead contact

Requests for further information and resources should be directed to and will be fulfilled by the lead contact, Zeina N. Al-Mahayri (zeina.almahairi@adu.ac.ae).

### Materials availability

This study did not generate new, unique reagents.

### Data and code availability

All allele-frequency data extracted from included studies and all pooled analyses generated in this work are provided in the main text and Supplemental Information (Tables S2, S3, and S4). This study did not generate new datasets or custom code. Additional details required to reproduce the analyses are available from the lead contact upon reasonable request.

## Acknowledgements

The authors acknowledge the researchers whose studies contributed to the body of pharmacogenomic data analyzed in this review. Their work has been essential in advancing the understanding of pharmacogenomic variation across Arab populations. The authors also thank Abu Dhabi University (ADU) and United Arab Emirates University (UAEU) for supporting the research environment that enabled the completion of this work.

## Author contributions

Conceptualization, Z.N.A.; methodology, Z.N.A. and B.R.A.; investigation, M.N.A., L.Q.K, S.M.A, A.A., and L.D.; writing – original draft, Z.N.A., M.N.A., L.Q.K, S.M.A, A.A., and L.D.; writing – review and editing, Z.N.A.; funding acquisition, B.R.A.; resources and supervision, Z.N.A and B.R.A.

## Declaration of interests

The authors declare no competing interests.

## STAR★Methods

### Key resources table

**Table:** 

REAGENT or RESOURCE	SOURCE	IDENTIFIER
**Deposited data**		

Extracted pharmacogenomic allele-frequency data from published studies	This study	Included in Supplemental Information (Tables S2, S3, and S4)

**Software and algorithms**		

R version 4.4.0	R Foundation for Statistical Computing	https://www.r-project.org/

**Other**		

gnomAD v4.0.1.	–	https://gnomad.broadinstitute.org/
ClinPGx (formerly PharmGKB)	–	https://www.clinpgx.org/
PharmVar	–	https://www.pharmvar.org/

### Experimental model and study participant details

This study is a systematic review/meta-analysis of published studies.

### Method details

This systematic review followed the Preferred Reporting Items for Systematic Reviews and Meta-Analyses (PRISMA) guidelines. The review protocol was submitted to the International Register of Systematic Reviews (PROSPERO) and registered under the number (CRD42023393633).^80^

#### Inclusion criteria

Studies performed genetic testing using a valid genotyping technique (e.g., conventional PCR-based genotyping, real-time PCR, Sanger sequencing, NGS, microarrays) on Arab individuals. Participants were self-identified Arabs or those originating from the listed Arab countries, given that the study explicitly reports allele frequencies of variants in the selected pharmacogenes. Pharmacogenetic variants are rarely associated with diseases.^81^ Hence, our inclusion criteria allowed for healthy and disease cohorts.

#### Exclusion criteria

Literature reviews and studies that did not report allele frequencies or genotypes were excluded. Studies that included a mixture of Arab and non-Arab populations without a discrete description of the frequencies were also excluded.

#### Information sources

The following four databases were searched: PubMed, Scopus, ScienceDirect, and Google Scholar, as well as two registries: ClinicalTrials.gov and the WHO International Clinical Trials Registry Platform (ICTRP).

#### Search strategy

A comprehensive search strategy was implemented using the names of pharmacogenes (Table 1-A) with clinical guideline annotations as per the PharmGKB list^82^ and the names of Arab countries identified as members of the Arab League (Table 1-B).

The selection of pharmacogenes in this review followed the PharmGKB list of genes with actionable clinical recommendations (Level 1A evidence), which was the authoritative source at the time we designed the review, prior to the migration of guideline annotations to ClinPGx. From the 22 Level 1A genes available in PharmGKB, we included 16 genes in our search. Six genes were excluded for predefined reasons. *CFTR* was excluded because its pharmacogenomic implications relate to ivacaftor responsiveness in individuals with cystic fibrosis; thus, the target population is diseased and fundamentally different from the general populations examined in this review. *HLA*-A and *HLA*-B were excluded because an extensive, recent, region-wide *HLA* meta-analysis in Arab populations has already been published,^79^ providing comprehensive allele-frequency representation that would not be enhanced by our approach. MT-*RNR1* was excluded because it is a mitochondrial gene, and mitochondrial inheritance patterns differ from the autosomal pharmacogenes examined in this review. *CYP3A4* was excluded because, although classified as Level 1A at the time, it lacked CPIC or DPWG actionable clinical prescribing recommendations for any medication, and it was also absent from older curated lists on which our protocol was initially based. Finally, *G6PD* was excluded because our preliminary extraction identified more than 120 studies, which is far more than for any other included gene, and including it would have disproportionately dominated the analysis. Additionally, several large-scale regional reviews already characterize its population distribution.^83^ The final gene panel, therefore, comprised 16 pharmacogenes with established actionable clinical guidance and relevance to population-level pharmacogenomics.

#### Data management

Each investigator was assigned a group of genes from Table 1-A and searched the databases for combinations of the gene and countries in Table 1-B (e.g., “*CYP2D6* Algeria”, “*CYP2D6* Bahrain”). A two-step identification process was conducted to ensure thoroughness by exchanging search terms between two investigators. Titles and abstracts were screened to retain relevant manuscripts.

#### PRISMA flow diagram

A PRISMA flow diagram^84^ was used to document the search and selection process. It illustrates the number of records identified, included, and excluded at each stage, along with reasons for exclusions.

#### Data extraction

The full texts of the included studies were retrieved. Information was extracted using a standardized Excel template, including [Sample size, Ethnic group, Clinical characteristics, Method used, Gene name, Variant ID, Variant frequency, Allele frequency, Key effect (if applicable), Drug and drug family (if applicable), Reference (Study link)]. Ethnicity information was extracted when explicitly reported; however, ethnicity-specific data were inconsistently available and reported in only a small number of studies, precluding systematic ethnicity-level analyses.Two separate investigators conducted data extraction twice on the same articles to ensure thoroughness and accuracy.

### Quantification and statistical analysis

#### Data synthesis

Data were synthesized using descriptive and quantitative approaches. For each pharmacogenomic variant, allele frequency data were summarized at the country level. When multiple independent studies reported data for the same variant within the same country, results were aggregated to generate pooled country-level estimates rather than treated as separate observations. Geographic maps were created to visualize pooled allele frequencies of selected clinically relevant star alleles across Arab populations.

#### Sample size threshold and aggregation of allele frequencies

To improve estimate precision and reduce the influence of very small cohorts, we applied a minimum sample-size threshold when generating country-level summary allele frequencies. For each variant, only studies with a cohort size of ≥50 individuals were included in the pooled frequency calculations and subsequent statistical analyses. Studies with smaller sample sizes were retained for qualitative description but did not contribute to pooled estimates. For each eligible study, we extracted genotype counts when available or, when only minor allele frequencies (MAF) were reported, we reconstructed allele counts by multiplying the MAF by twice the sample size (2N). Genotype counts were converted to allele counts as ALT = 2×(#homozygous alternate) + (#heterozygous), and REF = 2×(#homozygous reference) + (#heterozygous). For each (country, variant) pair, allele counts were then summed across all eligible studies to generate pooled ALT and REF counts. Pooled MAF for a given variant was calculated by dividing the total number of minor alleles by the total number of alleles (ALT/[ALT + REF]). These pooled MAFs were used for descriptive statistics, geographic mapping, and all downstream quantitative analyses.

#### Statistical comparison of allele frequencies with gnomAD-ME

To formally assess differences in allele frequencies between Arab populations and a global reference, we compared pooled country-level allele counts with the corresponding allele counts reported for the Middle Eastern subset of gnomAD (gnomAD-ME). Statistical analyses were performed for the set of 15 pharmacogenetically important variants selected for the clustering analysis, as these variants had sufficient cross-population data for valid comparisons. For each (country, variant) pair with adequate sample size, 2 × 2 contingency tables were constructed contrasting ALT and REF allele counts in the Arab country versus gnomAD-ME. Chi-square (χ^2^) tests were applied when all expected cell counts were ≥5; otherwise, Fisher’s exact tests were used. Resulting *p*-values were adjusted for multiple testing using the Benjamini–Hochberg false discovery rate (FDR) procedure. Statistical significance was defined as FDR-adjusted *p* < 0.05. Heatmaps illustrating allele frequency distributions across populations, hierarchical clustering, and pairwise correlation analyses were generated using pooled MAFs derived from aggregated allele counts. Variant-specific analyses (e.g., geographic maps and comparisons with gnomAD for individual variants) similarly relied on pooled country-level estimates when multiple studies were available, ensuring consistent weighting and comparability across all analyses. All statistical analyses and visualizations were conducted in R using the associated packages.

### Additional resources

Protocol registration Al-Mahayri et al., The Landscape of Pharmacogenomics Variations in Arabs: A systematic review, (2023). https://www.crd.york.ac.uk/prospero/display_record.php?RecordID=393633.

## Supplemental information

Table S1. Variants retrieved from the included studies listed per country

Table S2. Variants retrieved from the included studies listed per gene

Table S3. Variants used in pooled data analysis

Document S1. PRISMA checklist
